# The Shipibo Ceremonial Use of Ayahuasca to Promote Well-Being: An Observational Study

**DOI:** 10.3389/fphar.2021.623923

**Published:** 2021-05-05

**Authors:** Debora Gonzalez, Jordi Cantillo, Irene Perez, Maria Carvalho, Adam Aronovich, Magi Farre, Amanda Feilding, Jordi E. Obiols, José Carlos Bouso

**Affiliations:** ^1^ International Center for Ethnobotanical Education, Research and Services (ICEERS), Barcelona, Spain; ^2^ PHI Association, Barcelona, Spain; ^3^ Fundación BeckleyMed, Barcelona, Spain; ^4^ Catholic University of Portugal, Barcelona, Spain; ^5^ Medical Anthropology Research Center, Department of Anthropology, Philosophy and Social Work, University of Rovira I Virgili, Tarragona, Spain; ^6^ Clinical Pharmacology Unit, Hospital Universitari Germans Trias I Pujol and Institut de Recerca Germans Trias I Pujol (IGTP), Barcelona, Spain; ^7^ Department of Pharmacology, Therapeutics and Toxicoloy, Universitat Autònoma de Barcelona, Barcelona, Spain; ^8^ The Beckley Foundation, Oxford, United Kingdom; ^9^ Department of Clinical and Health Psychology, Faculty of Psychology, Autonomous University of Barcelona, Barcelona, Spain; ^10^ Department of Neurosciences and Behavior, Ribeirão Preto Medical School, University of São Paulo, Brazil

**Keywords:** well-being, quality of life, herbal medicine, Shipibo, ayahuasca, traditional medicine

## Abstract

Promoting well-being is one of the main goals to improve health in the world. We examined the well-being and quality of life over the course of one year in a sample that participated in an Indigenous Shipibo healing program where traditional healers work in a series of ayahuasca ceremonies. We also explored the role of decentering as a mediator of psychological well-being. Participants who attended the program responded to an online survey that included a Psychological Well-Being Scale; Oxford Happiness Questionnaire; The World Health Organization Quality of Life Spirituality, Religiousness, and Personal Beliefs scale; the WHO Quality of Life-BREF scale; and Decentering scale. Baseline (T0) and postassessment (T1) were completed by 200 individuals. Of these, 101 completed the follow-up assessment at three months (T2), 91 at 6 months (T3), and 94 at 12 months follow-up (T4) after leaving the center. ANOVA test was performed in a representative subsample to control the passing of time two months before attending the program (T-1). Pearson’s test was performed to examine the relationship between psychological well-being and decentering during the period of T0 and T1. A significant increase was observed in all the scales at all time points (*p* ≤ 0.01). The subgroup analysis performed in a representative subsample allowed us to infer that the significant differences in outcomes are due to the effect of their stay at the center and not the passing of time. We found a relationship between decentering and the improvement of psychological well-being (*r* = 0.57; *p* < 0.01). Our results suggest that the Indigenous Shipibo healing work with ayahuasca has value to improve long-term well-being and quality of life for Westerners.

## Introduction

In recent years, a growing interest in well-being, both in society in general as well as in mental health care, developed. This trend is reflected in the World Health Organization (WHO, 2001) definition of mental health as “a state of complete physical, mental and social well-being and not merely the absence of disease or infirmity” (WHO, 2006). Promoting well-being has become a principal goal for improving health in general among academics, health-care professionals, and policymakers alike (WHO, 2020a). Although there is no consensus on the definition of well-being, clinical research on this concept is rooted in two complementary schools of thought. The eudaimonic model postulates a psychological well-being that is achieved through the development of the human being’s potential. This is determined by the combination of six factors: autonomy, self-acceptance, environmental mastery, positive relations with others, purpose in life, and personal growth (Ryff, 1989). Alternatively, the hedonistic model defines well-being as a subjective state based on the pleasure and enjoyment of life, determined through three factors: life satisfaction, the presence of positive affect, and the absence of negative affect (Diener et al., 1999). In other words, hedonic construct deals exclusively with affect, measuring whether people are “feeling good”, but should be complemented with the eudaimonic construct that deals with personal-value fulfillment and meaningfulness, whether people are “doing well” or not (Michaelson et al., 2009).

One of the strategies to promote comprehensive human welfare at the international level has been to develop a strategic plan 2014–2023 to foster the appropriate integration, regulation, and supervision of traditional medicine (TM) (WHO, 2013). Traditional medicine is the sum total of the knowledge, skill, and practices based on the theories, beliefs, and experiences indigenous to different cultures—whether explicable or not—used in the maintenance of health, as well as in the prevention, diagnosis, improvement, or treatment of physical and mental illness (WHO, 2020b). Herbal medicines constitute the main component of traditional medicine, which has been used for thousands of years (WHO, 2010). Quality standards, safety, and efficacy of the herbal products are needed to be considered herbal medicines. However, governments and consumers are interested in more than herbal medicines and are now beginning to consider traditional medicine practices and practitioners and whether they should be integrated into health service delivery (WHO, 2020c).

Ayahuasca is an herbal concoction used for ritual and healing purposes since pre-Columbian times (McKenna, 1999). It is usually obtained by boiling the pounded stalks of the vine *Banisteriopsis caapi* (Spruce ex Griseb.) Morton and the leaves of the bush *Psychotria viridis* Ruiz & Pav. (Schultes, Hoffman, 1992). *B. caapi* contains *ß*-carboline alkaloids such as harmine, tetrahydroharmine (THH), and harmaline, that are reversible inhibitors of monoamine oxidase A (MAO-A), while *P. viridis* is rich in the tryptamine hallucinogen dimethyltryptamine (DMT), an agonist of serotonin (5-HT)1A/2A/2C and sigma-1 receptors (Riba et al., 2003; Fontanilla et al., 2009). Quantitative determination of other inorganic elements have displayed safe administration concentrations in 19 samples made from these plants collected from the União do Vegetal (Guimarães et al., 2020). Safety has been demonstrated by the long history of use, in observational studies on long-term users in ceremonial contexts (Grob et al., 1996; Halpern et al., 2008; Fabregas et al., 2010; Bouso et al., 2012; Bouso et al., 2015; Barbosa et al., 2016; Jiménez-Garrido et al., 2020), and in the laboratory (Mello et al., 2019; dos; Santos, 2011; Hamill et al., 2019). Moreover, open trials and randomized trials have shown the efficacy of ayahuasca as a rapid antidepressant (Osorio et al., 2015; Sanches et al., 2016; Palhano-Fontes et al., 2019). Observational studies show a higher level of well-being in long-term users of ayahuasca (Bouso et al., 2012; Lawn et al., 2017; Ona et al., 2019) and lifestyle modifications (Grob et al., 1996).

Today, interest in ayahuasca is a global phenomenon. Outside its native Amazonian habitat, ayahuasca has a presence in dozens of countries, including other parts of South America, North America, Europe, Australia, New Zealand, and some parts of Asia (Tupper, 2008). This expansion has happened through three ways: religions that use ayahuasca as a sacrament; the psychonautic use of ayahuasca brew by Western people and neo-ayahuasqueros; and the cross-cultural vegetalismo or Indigenous-style ayahuasca healing ceremonies conducted in an often overtly commodified way for non-Indigenous clients both in the Amazon and abroad (Tupper, 2009). In these traditions, aspiring ayahuasqueros go through an extended and difficult period of training involving demanding dietary and behavioral restrictions—although real mastery is acknowledged to take decades or a lifetime (Langdon, 1979).

However, the absence of international regulation of ayahuasca use poses a number of serious risks, both for Indigenous peoples and for the international community in general. On the one hand, Indigenous people see the desecration and cultural appropriation of their knowledge, symbols, stories, ceremonies, dances, and songs (Zuluaga, 2000; Tupper, 2009), and fear that their ancestral heritage will be changed or lost. On the other hand, Westerners who take ayahuasca have no guarantees about the composition of the ayahuasca, unwanted pharmacological interactions, exposure to unreliable information, and unqualified and unethical practitioners (Labate and Araujo, 2004; Qui, 2013; Kavenská, Simonvá, 2015).

Since these risks underlie all traditional medicines using herbal medicines, the World Health Organization has promoted international recognition of traditional medicine by regulating the products, practices, and practitioners (Callaway et al., 1999). It has included—for the first time—diagnostic criteria based on traditional Chinese medicine in the 11th edition of the International Classification of Diseases (ICD-11) (WHO, 2018). This effort has a reciprocal impact on the safety of doctors and patients, both in the countries of origin and in the countries that import medicinal practices and products. For example, acupuncture is covered by public and private insurances in different European countries by accredited professionals favoring the exercise of good practices among professionals and patients. Knowledge-based policies are crucial for the integration of traditional medicine into national health systems, and research should be prioritized and supported in order to generate knowledge (WHO, 2013). Randomized placebo-controlled clinical trials provide a high internal validity but low external validity. Therefore, clinical trials in the laboratory have to be combined with observational research to study how these medical systems are working in the real world.

### Traditional Context

This article presents the results of a longitudinal observational study evaluating various parameters related to the psychological, subjective, spiritual well-being, and quality of life of Western people attending retreats at the Temple of the Way of Light in Iquitos, Peru (Temple of the Way of Light, 2016). Ceremonies are run by five male and female Shipibo *onanyabo* (the plural form of the Shipibo term for an ayahuasca practitioner), supported by two Western facilitators. Although these ceremonies are rooted in the traditional Shipibo medical system, they have been adapted to working with Western participants. According to the Shipibo *onanyabo*, their work over the course of the series of ceremonies follows a coherent and structured progression, with the first ceremony being a diagnostic session. As the ceremony begins, the first *icaro* that they perform is meant to create a safety structure around the ceremonial space, with the intention of protecting the participants from energetic interferences from the outside environment and between participants. Following Dobkin de Rios (1984), *icaro* is the generic name given to the medicine songs used by the curanderos of the Peruvian Amazon Basin (for a deeper review regarding *icaros*, see Bustos, 2008). During the initial diagnostic session, the *onanyabo* aims to detect energetic blockages in the participants, “scanning” their bodies with the help of the *icaro*.

The following ceremonies, according to the *onanyabo*, aim to cleanse and clear “dense energies” that participants may carry. Although the Shipibo does not necessarily make the same conceptual connections, Western facilitators often understand these dense energetic knots to correlate with psychosomatic and emotional imprints leftover from individual and *trans*-generational traumas, repressed emotions, or self-sabotaging unconscious views that are rooted in past difficult life episodes, particularly childhood experiences. However, diagnosis is also a continuous process throughout the cleansing and clearing process, reaching into and revealing deeper layers of energetic and spiritual ways of being. When “heavy energies” are uncovered, the *onanyabo* claim to use *icaros* to disconnect and dispel them within the individual’s body, sending the “energies” back into the earth. By clearing dense energies from the past, participants become “unstuck” and often significantly transform their relationship with themselves, others, and the world in general.

Once the cleansing and clearing have been done, a culminating last ceremony occurs. The *onanyabo* focuses on sealing in the “spiritual surgery” and aligning the body, mind, and spirit, together with the perceived energetic structure of each participant, protecting the energetic and physical body while illuminating the pathway forward.

In addition to the *icaros*, the Shipibo also works with *Nicotiana rustica*, commonly known as Mapacho, Aztec, Brazilian, or strong tobacco, as an important plant ally in the ceremony. They occasionally blow smoke on the participants to transmit the *icaros*, cleanse heavy energies, and maintain protections. In addition, several plants and flowers in the form of steam baths and flower baths are used outside of the ceremonies in order to cleanse the energies that are being pulled out by the *icaros*. Several perfumes, such as *agua florida* or *pusanga*, are also used to help the person “blossom” and attract positivity into their lives.

The present study investigated the long-term effects of ayahuasca in the well-being of participants from a holistic view. The primary outcome of this study was psychological well-being. Secondary variables were subjective well-being, spiritual well-being, quality of life, and decentering. We hypothesized that the psychological well-being would be improved after drinking ayahuasca in this traditional healing work context still maintained after a one-year follow-up. Second, we hypothesized that subjective well-being, spiritual well-being, quality of life, and decentering would also improve after drinking ayahuasca. Given that previous studies have shown that decentering is a psychological process underlying ayahuasca’s effects (Soler et al., 2016; Franquesa et al., 2018; Gonzalez et al., 2020; Murphy-Beiner, Soar, 2020) and that this psychological process mediates psychological well-being (Park and Park, 2017; Carmody et al., 2009), we finally hypothesized that changes in psychological well-being after being at the center will be mediated by changes in decentering.

## Materials and Methods

This study is part of a broader research project that aims to prospectively assess the long-term effects of ayahuasca on psychopathological symptoms in different subsamples with depression, anxiety, posttraumatic stress disorder, and grief. Grief data have been published in a previous study (Gonzalez et al., 2020). In the present article, only the data for the subsample that have not been previously diagnosed with a mental disorder and do not suffer from any of those psychopathological symptoms have been included and analyzed. The study was approved by the Ethics Committee on Animal and Human Experimentation of the Autonomous University of Barcelona (UABCEEAH Barcelona, Spain) and was conducted in accordance with the Declaration of Helsinki. Participants signed an online informed consent and were not financially compensated for their participation.

### Participants and Procedure

Participant enrollment was carried out between 2015 and 2017. During 2018, the follow-up period of one year was concluded. Participants were invited to participate in the study through the Web site of the Temple of the Way of Light (Temple of the Way of Light, 2016) after having booked their stay at the center. This center offered retreats of 9, 12, or 30 days, where participants drank ayahuasca between 1 and 12 times in a ceremonial context under the guidance of Shipibo *onanyabo*.

A total of 200 participants were eligible for the analysis. The screening process involved two stages. First, to be accepted by the center, individuals had to be 18 years or older. Moreover, people with previous clinical disorders (psychosis, depersonalization, and mania), people taking certain medications (i.e., MAOIs or SSRIs), subjects with a heart condition, those with chronic high blood pressure, and pregnant women were not accepted (Temple of the Way of Light, 2019). Second, participants must respond to a short online questionnaire including the following questions: 1) Please indicate the exact date when you will attend to the retreat; 2) Please mark “yes,” “uncertain,” or “no” to the following questions: Are you currently going through a period of (b.1) depression, (b.2) anxiety, (b.3) posttraumatic stress, or (b.4) grief related to the death of a loved one; 3) Have been diagnosed with a mental disorder in the past?, and 4) Can you understand with ease everything you read in the English language?

Participants were included in the study if they were not currently experiencing depression, anxiety, posttraumatic stress, and grief symptoms related to the death of a loved one; if they have not been diagnosed with certain mental disorders in the past; and if they affirmed having adequate knowledge of the English language. Once participants were included in the study, assessments were done 15 days before attending the retreat (T0) and 15 days (T1), three months (T2), six months (T3), and 12 months (T4) after leaving the center. Only participants who responded baseline (T0) and postassessment (T1) were finally included in the analysis. Participants who signed informed consent more than two months before attending the center were invited to respond to an additional assessment two months before entering the center (T-1) in order to control for the effect of time on outcomes in a subgroup.

All questionnaires were administered online using LimeSurvey (version 1.92.), which allowed for the collection and preservation of the data for confidentiality on a secure server that is accessible only to the researchers with a secure password.

### Measures

A series of questionnaires were administered to the subjects.

### Psychological Well-Being

Psychological Well-Being Scales (PWBS) (Ryff, 1989) is a 42-item Likert-type scale (1 = completely disagree, 6 = completely agree), which measures psychological well-being through scales of Self-Acceptance, Positive Relationships, Autonomy, Environmental Mastery, Personal Growth, and Purpose in Life. Scores in each domain range from 6 to 42 where higher scores indicate greater levels of well-being. In the current study, the sum of the score ratings across the six dimensions was added including an overall PWBS measure (*α* = 0.89), with higher scores reflecting higher PWBS.

### Happiness

Oxford Happiness Questionnaire (Hills and Argyle, 2002) measures happiness or subjective well-being (Medvedev and Landhuis, 2018). A series of statements about happiness are given and the participants indicate their degree of agreement with each one. In psychometric terms, it consists of 29 items, and the participants assess the extent to which they form part of their experiences. It employs a Likert scale (1 = I totally disagree, 6 = I totally agree). Reliability for this scale was found to be 0.91.

### Spiritual Well-Being

WHO Quality of Life Spirituality, Religiousness, and Personal Beliefs (WHOQOL-SRPB BREF) (WHOQOL SRPB Group, 2006) is a 32-item Likert scale (1 = not at all, 5 = an extreme amount), covering quality-of-life aspects related to spirituality, religiousness, and personal beliefs. It is divided into eight scales: Spiritual Connection, Meaning and Purpose in Life, Experiences of Awe and Wonder, Wholeness and Integration, Spiritual Strength, Inner Peace, Hope and Optimism, and Faith. Each facet score is derived by averaging the score obtained from the responses to the four questions comprising that particular facet. The domain score is calculated by adding the scores of the facets. Cronbach’s *a* for the SRPB facets has been found to be strong, ranging from 0.77 (meaning and purpose in life) to 0.95 (faith), whereas Cronbach’s *a* for the complete WHOQOL-SRPB instrument is excellent (*α* = 0.93).

### Quality of Life

WHO Quality of Life-BREF (WHOQOL-BREF) (WHOQOL Group, 1998) is a shorter version of the original WHOQOL-100 (Power et al., 1999). This questionnaire was designed to assess quality of life in four domains: Physical Health, Psychological Health, Social Relationships, and Environment. It comprised 24 items, scored on a 4–20 scale, with higher scores indicating a better quality of life. For the purpose of this study, only the first three scales were used. The scale has been shown to have adequate psychometric properties with Cronbach’s coefficient *α* values ranging from 0.68 to 0.82 (61).

### Decentering

EQ-Decentering (Fresco et al., 2007): this scale assesses decentering, defined as the ability to observe one’s thoughts and feelings in a detached manner. This is an 11-item, self-report measure of decentering. Items are rated on a 5-point Likert scale (1 = never to 5 = always). The original EQ showed a high-reliability coefficient with Cronbach’s *α* value of 0.90.

### Ayahuasca Samples Analyses

Two ayahuasca samples collected during the participants’ enrollment were analyzed (Gonzalez et al., 2020). The ayahuasca was prepared by boiling the stems of *Banisteriopsis caapi* (Spruce ex Griseb.) Morton, rich in harmine, tetrahydroharmine, and harmaline, combined with the leaves of *Psychotria viridis* Ruiz & Pav., rich in DMT. Analyses were carried out by Energy Control (energycontrol-international.org) using liquid chromatography–mass spectrometry (LC–MS). One ayahuasca sample contained 2 mg/ml DMT, 2 mg/ml of harmine, 0.37 mg/ml of harmaline, and 1 mg/ml of tetrahydroharmine. The other sample contained 2 mg/ml DMT, 2 mg/ml of harmine, 0.65 mg/ml of harmaline, and 2 mg/ml of tetrahydroharmine. No other psychoactive compounds were detected.

### Statistical Analyses

Baseline sample characteristics, setting variables, persistent adverse effects, benefits of ayahuasca over time, and risk’s perception of the ayahuasca use in the participants’ original countries were analyzed descriptively. Changes in primary and secondary outcome variables between each time point and baseline were tested using Student’s paired two-sided T-test or the Wilcoxon-paired test, as appropriate. Bonferroni corrections were applied to correct for multiple comparisons. A 95% CI for the mean of the primary outcome at all-time point assessments was included to further clarify plausible values. Effect sizes were calculated using Cohen’s *d*. Effect sizes were defined as large up to 0.8 (*d* > 0.8), medium (*d* > 0.5), and small (*d* ≤ 0.2) (Cohen, 1992). Pearson’s linear correlation coefficient was used to examine the relationship between Decentering (EQ-Decentering) and Psychological Well-Being Scale (PWBS). Correlation coefficients between 0.30 and 0.70 were considered moderate.

Additional subgroup analysis was performed separately for patients who responded to T-1, T0, and T1 assessments. We used the chi-square test for categorical variables and Fisher–Snedecor for continuous variables to evaluate homogeneity between the group that completed the follow-up at T1 and a subgroup that completed an additional assessment at T-1. Subgroup analyses were conducted using repeated measures analysis of variance (ANOVAs) with post hoc Bonferroni correction in order to examine differences in primary and secondary outcomes between the periods of T-1 to T0 and T1 to T0. Effect sizes were calculated using Cohen’s d.

In this study, *p* values <0.05 were considered statistically significant. Statistical analysis was performed using Statistical Package for the Social Sciences (SPSS for Windows, version 20).

## Results

### Participants

**FIGURE 1:**
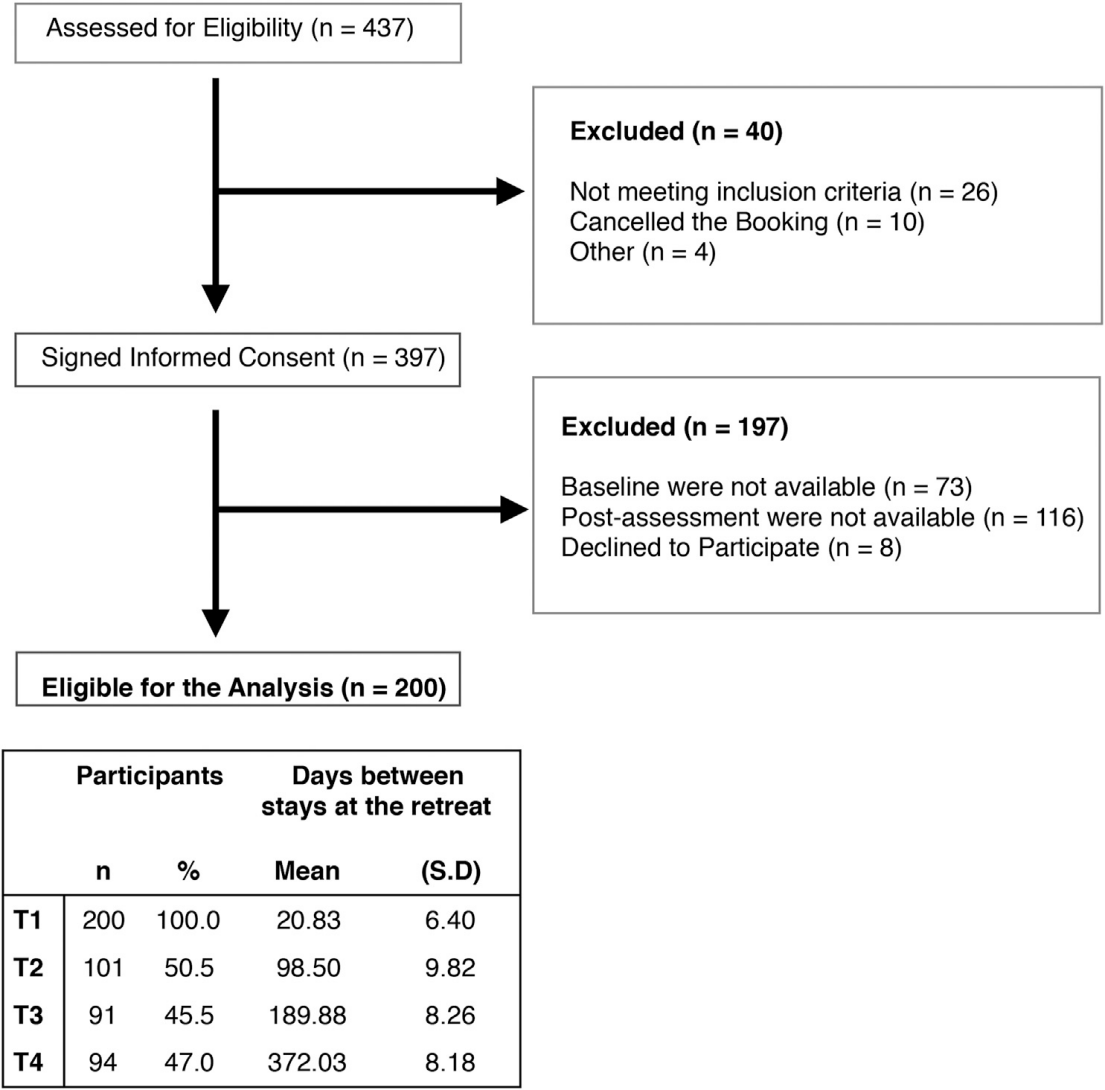
Participant flow throughout the study.

Out of 437 participants who were assessed for eligibility, 90.8% of participants signed the informed consent form. However, 18.4% of participants were excluded because baseline assessment (T0) was not available, 36.7% were excluded because postassessment was not available (T1), and 2% expressed a desire to abandon the study. Finally, 200 participants were eligible for the analysis, having completed the baseline and postassessment. Overall, the rate of participants not responding to the assessment at T2 was 49.5%, at T3 was 54.5%, and 53% at T4 (Figure 1).

### Baseline Sample Characteristics

The demographic characteristics of participants are summarized in Table 1. The motivations for attending the retreat were spiritual growth (66.7%) and personal development (32.4%). None of the participants indicated a therapeutic motivation or recreational purpose, such as drug tourism. Of the overall sample, 65.5% had never used ayahuasca before. Of the remaining 34% of the sample (*n* = 68), 9.5% drank ayahuasca less than 4 times, 13% between 4 and 10 times, and 11.5% more than 10 times.

**TABLE 1:** Demographic characteristics of participants.

		*N* = 200	
Sociodemographic characteristics		*N*	%
Male		104	52.3
Age in years^a^		41.84 (11.69) [20; 69]	
Educational level			
Primary school		4	2.0
Secondary/High school		20	10.0
College/University		169	84.5
Other		7	3.5
Race			
Caucasian		178	89.0
Hispanic		9	4.5
Asian		6	3.0
Others		7	3.5
Country			
United States of America		57	35.4
United Kingdom		29	18.0
Australia		16	9.9
Canada		18	11.2
Germany		9	5.6
Other		32	19.9
Marital status			
Single		93	46.7
Married		35	17.6
Living as married		30	15.1
Divorced or separated		41	20.6
Employed		146	73.0
Nonreligious		190	95.0

a Data are expressed as mean (SD) [range].

### Setting Variables

Participants attended an average of 14.93 (SD = 7.58) days at the center (range 9–31), where they participated in an average of 6.21 (SD = 1.57) ayahuasca ceremonies (range 1–12). Of the 200 participants who responded at T0–T1, 54% perceived ayahuasca as the most relevant factor in the evolution of their well-being during their stay at the center, followed by the work of the Shipibo healers (36%), being with other guests (6%), the experience of being in the jungle (2.5%), and having attended other alternative activities, such as yoga classes (1.5%).

### Main Outcome Analyses

The results for outcome variables at all assessment points (T0, T1, T2, T3, and T4) are presented in Table 2. Psychological Well-Being Scales Total score was significantly increased from baseline to all point times follow-up assessment (T1, T2, T3, and T4: *p* < 0.01). Calculating effect sizes according to Cohen’s method, we found a high effect size at all assessment points (T1 = 0.89, 95% CI = 0.72–1.04; T2 = 0.91, 95% CI = 0.67–1.11; T3 = 1.12, 95% CI = 0.89–1.35; and T4 = 0.82, 95% CI = 0.56–1.07).

**TABLE 2:** Outcome of repeated measures at baseline (T0) and each time point during follow-up. Values are given as means differences (with standard deviations).

		T1–T0	T2–T0	T3–T0	T4–T0
Measures		Mean (SD) Sig.^a^ ^†^	Mean (SD) Sig.^b^ ^†^	Mean (SD) Sig.^c^ ^†^	Mean (SD) Sig.^d^ ^†^
		*N* = 200	*N* = 101	*N* = 91	*N* = 94
PWBS					
Autonomy		2.83 (4.93)**	3.12 (4.73)**	3.16 (4.06)**	3.05 (4.38)**
Environmental mastery		2.39 (3.87)**	2.56 (4.11)**	2.63 (3.55)**	2.43 (4.05)**
Personal growth		1.69 (3.97)**	1.53 (3.94)**	1.56 (3.58)**	1.91 (4.07)**
Positive relations		2.63 (3.85)**	2.69 (4.35)**	2.62 (4.02)**	2.44 (4.48)**
Purpose in life		3.66 (5.39)**	3.21 (5.54)**	4.43 (5.84)**	3.82 (6.29)**
Self-acceptance		3.93 (5.06)**	3.91 (5.05)**	4.26 (4.59)**	3.87 (5.91)**
Total		16.88 (19.0)**	16.75 (18.49)**	18.73 (16.77)**	16.43 (19.92)**
WHOQOL-BREF					
Physical health		1.39 (1.83)**	1.11 (1.74)**	1.15 (1.68)**	1.22 (1.97)**
Psychological health		2.03 (2.11)**	1.63 (2.34)**	1.78 (1.89)**	1.68 (2.39)**
Social relationships		2.14 (2.72)**	1.81 (2.85)**	2.31 (2.70)**	2.10 (3.08)**
Oxford happiness		0.52 (0.68)**	0.43 (0.75)**	0.54 (0.60)**	0.50 (0.73)**
EQ-decentering		5.87 (6.03)**	4.30 (4.94)**	4.48 (5.16)**	4.65 (5.37)**
WHOQOL-SRPB					
Spiritual connection		0.52 (0.97)**	0.42 (1.04)**	0.67 (1.03)**	0.70 (1.04)**
Meaning of life		0.45 (0.69)**	0.31 (0.72)**	0.47 (0.66)**	0.45 (0.72)**
Experience of awe		0.55 (0.76)**	0.48 (0.71)**	0.71 (0.78)**	0.66 (0.74)**
Integrity and integration		0.46 (0.63)**	0.38 (0.60)**	0.47 (0.55)**	0.45 (0.57)**
Spiritual strength		0.43 (0.60)**	0.26 (0.64)**	0.29 (0.49)**	0.22 (0.64)**
Inner peace		0.50 (0.72)**	0.39 (0.78)**	0.56 (0.67)**	0.49 (0.69)**
Hope and optimism		0.68 (0.72)**	0.47 (0.75)**	0.53 (0.67)**	0.53 (0.74)**
Faith		0.56 (0.58)**	0.48 (0.62)**	0.48 (0.54)**	0.50 (0.59)**
Total		0.52 (0.54)**	0.39 (0.55)**	0.53 (0.50)**	0.49 (0.55)**

^†^Bonferroni correction is performed.

a *p* value calculated by paired sample *T-*test from T0 to T1 (*N* = 200).

b *p* value calculated by paired sample T-test from T0 to T2 (*N* = 101).

c *p* value calculated by paired sample T-test from T0 to T3 (*N* = 91).

d *p* value calculated by paired sample T-test from T0 to T4 (*N* = 94).

Asterisks indicate *p* values: **p < 0.01 in the paired sample T-tests.

PWBS, Psychological Well-Being Scales; WHOQOL-BREF, World Health Organization Quality of Life; WHOQOL-SRPB, World Health Organization Quality of Life Spirituality, Religiousness, and Personal Beliefs.

Subjective well-being also showed a significant improvement at all-point assessments (T1, T2, T3, and T4: *p* < 0.01), showing a moderate effect size at T1 = 0.76, 95% CI = 0.62–0.90; T2 = 0.58, 95% CI = 0.37–0.77; and T4 = 0.69; 95% CI = 0.45–0.90. Unexpectedly, the effects size was higher at T3 = 0.91 95% CI = 0.68–1.12.

Similarly, the WHOQOL-SRPB showed a significant improvement on the total score for all assessments (T1, T2, T3, and T4: *p* < 0.01), showing a high effect size at T1 = 0.96, 95% CI = 0.80–1.11; T3 = 1.05, 95% CI = 0.83–1.725; and T4 = 0.90, 95% CI = 0.67–1.11. The effect size at T2 was medium. T2 = 0.71, 95% CI = 0.52–0.90.

The WHOQOL-BREF questionnaire also showed significant improvements in physical health, psychological health, and social relationship scales after the retreat (T1: *p* < 0.01). The effect size was larger for the psychological health scale (T1 = 0.96, 95% CI = 0.81–1.10; T2 = 0.70, 95% CI = 0.49–0.88; T3 = 0.94, 95% CI = 0.75–1.13; and T4 = 0.70, 95% CI = 0.51–0.87).

Finally, decentering significantly improved after the retreat (T1: *p* < 0.01) and at all follow-up assessments (T2, T3, and T4: *p* < 0.01). Decentering also showed a high-effect size at all-time point assessments (T1 = 0.97, 95% CI = 0.82–1.12; T2 = 0.87, 95% CI = 0.66–1.06; T3 = 0.87, 95% CI = 0.63–1.09; and T4 = 0.87, 95% CI = 0.61–1.10).

The improvements achieved in the key variables after the retreat remained stable at 12 months, with no significant differences observed between T4 and T1, except in spiritual strength (T4–T1 = −0.20 (0.57); *p* = 0.001) (Supplementary Table S1).

Persistent benefits and adverse effects derived from the ayahuasca experiences at the center are shown in Table 3.

**TABLE 3:** Adverse effects and benefits of ayahuasca at each time point during follow-up.

			T1		T2		T3		T4	
			*N* = 200		*N* = 101		*N* = 91		*N* = 94	
			n	%	N	%	n	%	n	%
Persistent benefits of ayahuasca^a^										
No			6	3.0	11	10.9	3	3.3	6	6.5
Yes			194	97.0	90	89.1	87	96.7	87	93.5
Physical health			95	47.5	43	42.6	33	36.7	36	38.7
Mental health			146	73.0	57	56.4	63	70.0	65	69.9
Personal well-being			175	87.5	70	69.3	76	84.4	77	82.8
Social relationships			126	63.0	58	57.4	51	56.7	46	49.5
Spiritual			164	82.0	78	77.2	77	85.6	76	81.7
Lifestyle			118	59.0	42	41.6	43	47.8	42	45.2
Persistent adverse effects of ayahuasca^a^										
No			195	97.5	100	99.0	91	100.0	94	100.0
Yes			5	2.5	1	1.0	0	0.0	0	0.0
Physical health			3	1.5	1	1.0	0	0.0	0	0.0
Mental health			1	0.5	0	0.0	0	0.0	0	0.0
Personal well-being			0	0.0	0	0.0	0	0.0	0	0.0
Social relationships			1	0.5	0	0.0	0	0.0	0	0.0
Spiritual			0	0.0	0	0.0	0	0.0	0	0.0
Lifestyle			1	0.5	0	0.0	0	0.0	0	0.0

a Multiple-choice response.

### Correlation Analyses

The significant increase in decentering between T0 and T1 was found to be associated with the significant increase in the Psychological Well-Being Total Scale between T0 and T1 (*r* = 0.53; *p* < 0.01).

### Subgroup Analyses

Of the 200 participants who completed the baseline and postassessment (T0 and T1), a subgroup of 42 participants who completed an additional assessment two months prior to the retreat (T-1) were eligible for the subgroup analysis (ANOVA). The homogeneity of the groups was tested by comparing key variables (baseline characteristics, primary and secondary outcome measures). Significant differences were found only in people with a university degree (control subsample = 87.3%; subsample = 73.8%; *p* = 0.031). No other significant differences were found in primary and secondary outcomes measures (*p* > 0.05).

**FIGURE 2:**
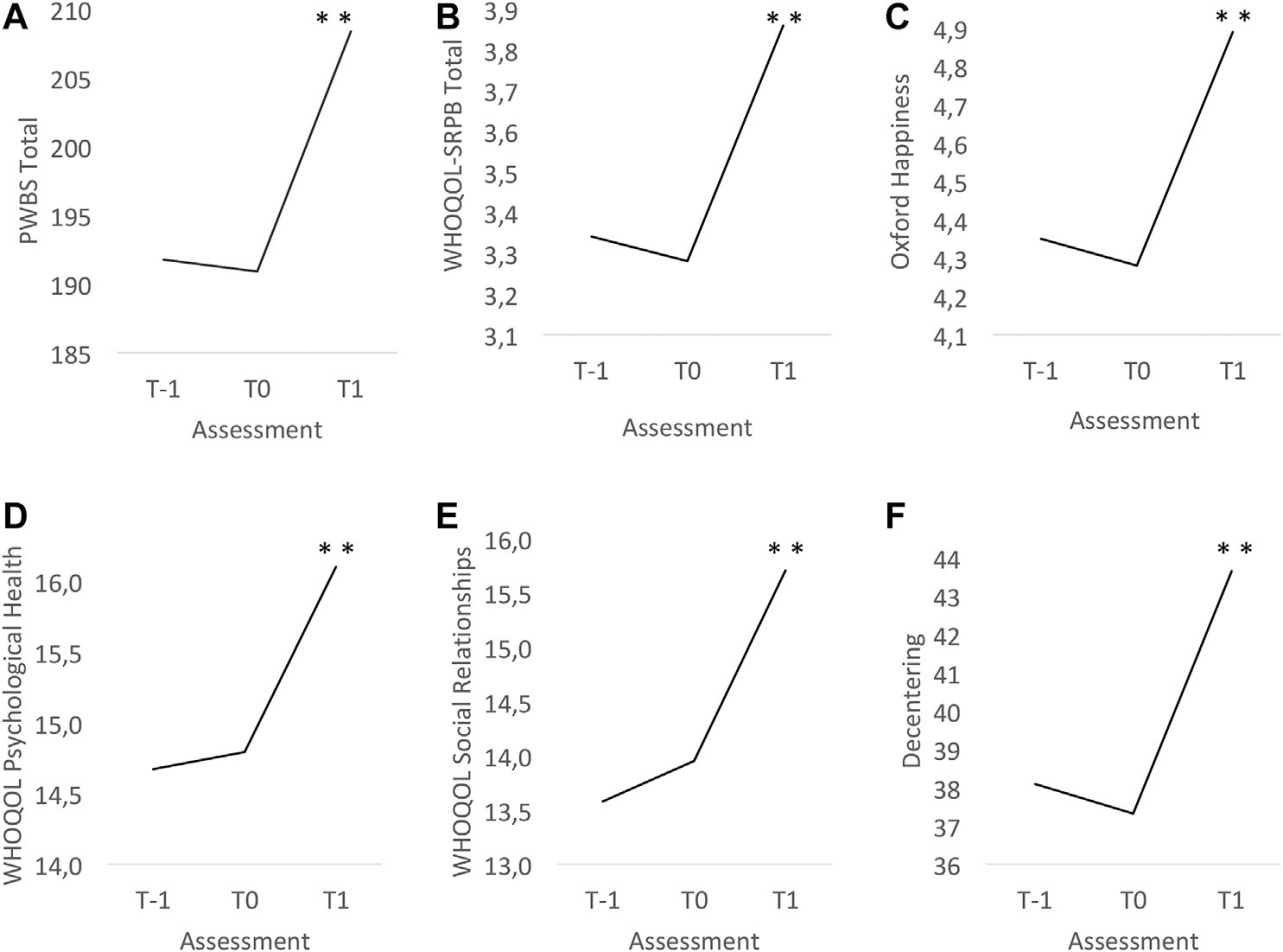
Graphs of the mean values (dots) of primary and secondary outcomes at T-1, preassessment (T0) and postassessment (T1). **(A)** Psychological Well-Being Scales: Total Scale. **(B)** WHO Quality of Life Spirituality, Religiousness, and Personal Beliefs (WHOQOL-SRPB): Total Scale. **(C)** Oxford Happiness Questionnaire. **(D)** World Health Organization Quality of Life-BREF (WHOQOL-BREF): Psychological Health Scale. **(E)** World Health Organization Quality of Life-BREF (WHOQOL-BREF): Social Relationship Scale. **(F)** EQ-Decentering Scale. *p value < 0.05. **p value < 0.01. T-1 = Two months before entering the center; T0 = 15 days before entering the center; T1 = 15 days after entering the center. PWBS, Psychological Well-Being Scales; WHOQOL-SRPB, World Health Organization Quality of Life Spirituality, Religiousness and Personal Beliefs; WHOQOL-BREF, World Health Organization Quality of Life.

The average number of days registered in the subsample between T-1 and T0 was 44.29 (SD = 5.42), and between T0 and T1, it was 46.0 (SD = 9.13). Mean values of primary and secondary outcomes in the subgroup analysis are presented in Figure 2.

ANOVA revealed a significant main effect of time on the PWBS Total [F (1.6; 58.6) = 26.84, *p* < 0.001]. Post hoc tests showed a significant improvement only between T1 and T0 [17.49 (SD = 18.98); *p* < 0.001] and not between T0 and T-1 [−0.88 (SD = 11.62); *p* > 0.999]. These results indicate that the reduction in the total score of psychological well-being was only observed during participants’ stays at the center. Effect size was large (0.92; 95% CI, 0.62–1.22).

Similarly, ANOVA revealed a significant main effect of time on secondary variables during participants’ stays at the center (Figure 2). Oxford Happiness Questionnaire [*F* (2; 82) = 26.14; *p* < 0.001] shows an improvement only between T1 and T0 [0.61 (SD = 0.61); *p* < 0.001] and not between T0 and T-1 [−0.07 (SD = 0.55); *p* > 0.999]. The effect size between T1 and T0 was large (0.99; 95% CI, 0.66–1.31).

Total Scale of WHOQOL-SRPB [*F* (1.4; 57.3) = 49.02; <0.001] shows the improvement only between T1 and T0 [0.58 (SD = 0.45); *p* < 0.001] and not between T0 and T1 [−0.06 (SD = 0.26); *p* = 0.504). The effect size between T1 and T0 was large (1.29; 95% CI, 0.93–1.65).

The subscales of the WHOQOL-BREF show a similar pattern, being Psychological Health Scale improved [*F* (1.7; 71.3) = 18.30; *p* < 0.001; *d* = 0.83; 95% CI, 0.53–1.13], only between T1 and T0 [1.31 (SD = 1.58); *p* < 0.001] and not between T0 and T1 [0.12 (SD = 1.48); *p* > 0.999], and Social Relationships Scale improved [*F* (2; 80) = 22.00; *p* < 0.001; *d* = 0.79; 95% CI, 0.44–1.15] only between T1 and T0 [1.76 (SD = 2.22); *p* < 0.001] and not between T0 and T1 [0.37 (SD = 1.80); *p* = 0.574].

Finally, the significative increase in decentering [*F* (1.4; 56.6) = 29.90; *p* < 0.001] is observed only between T1 and T0 [6.32 (SD = 6.68); *p* < 0.001) and not between T0 and T1 [−0.78 (SD = 3.32); *p* = 4.111]. Effect size between T1 and T0 was large (0.95; 95% CI, 0.65–1.26).

### Risk’s Perception of the Ayahuasca Use in the Participants’ Original Countries

At T2, 92.5% of the sample wished to continue taking ayahuasca in the future. However, only 14.9% drank ayahuasca between 1 and 3 times, 11.7% between 4 and 10 times, and 8.5% more than 10 times at T4. This means that 64.9% of the sample didn’t drink ayahuasca over the 12 following months after leaving the center.

Nevertheless, 92% of the sample affirmed to be exposed to several risks in taking ayahuasca in their country of origin, such as legal charges (71.3%), health problems due to the lack of guarantees regarding the composition of ayahuasca (40.8%), or the disability to consult with professional experts in case of adverse effects (26%). Moreover, 40.3% of the sample affirmed risks related to their personal safety not being able to guarantee that the person administering ayahuasca follows a professionally regulated practice and complies with a code of ethics. Finally, more than a half of the sample (59.7%) affirmed that ayahuasca should be practiced by Amazonian healers or practitioners with comprehensive traditional training.

## Discussion

In this prospective, observational non-controlled study, we examined the long-term impact that ayahuasca, used in a ceremonial Shipibo context, can have on the integral well-being of the individual. Participants who have completed the evaluations throughout the year show a significant increase in psychological well-being, subjective well-being, spiritual well-being, and quality of life after the stay in the retreat. These outcomes are confirmed by the reports of most of the participants who affirmed sustained benefits on personal well-being over one year, followed by spiritual benefits. The subgroup analysis performed in a representative subsample allowed us to infer that the significant differences in outcomes are due to the effect of their stay at the center and not the passing of time.

Well-being is a relevant worldwide factor directly linked to the prevention of mental disorders as well as to the promotion of mental health (Huppert, 2009). In fact, well-being is a highly complex construct that entails physical, mental, and social well-being (WHO, 2001). The results presented in this article show a significant and sustainable long-term improvement in well-being from a holistic view, including the physical and social relationships quality of life in addition to psychological, subjective, and spiritual well-being. These results are in line with several studies that have observed a high level of well-being in people who take ayahuasca regularly in ritual communitarian context (Ona et al., 2019), and in religious ceremonies (Bouso et al., 2012; Bouso et al., 2015; Barbosa et al., 2016). Ayahuasca users have shown even higher well-being than those who had taken classic psychedelics such as LSD or Psilocybe mushrooms (Lawn et al., 2017; Griffits et al., 2019), especially in life satisfaction, social relationships, spiritual awareness, and attitudes about self and life (Griffits et al., 2019). Since LSD or mushrooms are not usually used in a ritual context, these results lead us to consider the role of the ceremony in the positive and long-term impact on the well-being of the Western people (Cooper, 1987). Kaptchuk (2002) argues that the alternative medicine used in ritual settings may administer an especially large dose of “performative efficacy,” which relies on the power of belief, imagination, symbols, meaning, expectation, persuasion, and self-relationship. These factors could be understood in biomedical science as the placebo effect or what Miller and Kaptchuk (2008) call “contextual healing.” Indeed, in our sample, 36% of the participants affirmed that the work of the Shipibo healers was the most important factor in the improvement of their well-being, even more than ayahuasca itself. However, as it has been explained in the introduction, Shipibo healers claim to be performing a specific medical intervention that is not explicable by our Western, materialist worldview, probably because of our epistemological and perceptual biases (Turner, 1992; Walsh, 1990). Expanded science (Lorimer, 2019) is necessary to establish the extent to which the medical intervention of the healers could be conceived as performative contextual healing or whether it is a qualified and replicable medical practice rooted in indigenous knowledge and the underlying ontologies.

The effect size is high in all PWBS total assessments, self-acceptance being the subscale which presents a higher improvement after the retreat and follow-up assessments. Self-acceptance is defined as a positive attitude toward oneself that entails a regulation process of the self-concept, which is one of the most significant and powerful regulators of behavior and affect (Markus and Wurf, 1987). Distortions in the experience of one’s “self” are central to the psychedelic experience, such as in the “ego-dissolution experiences” (Nour et al., 2016). However, following Gergen (1985), self is also a construct cocreated by culture, societies, and individuals at a particular time and place, which is constituted in relationship with others. This implies that beyond the phenomenological experience with ayahuasca, the reflection process of the experience during the integration sessions where people are able to share in a collective with their own subculture could allow for the organic emergence of new meanings about the self that could be easier to accept. Indeed, several authors describe how ayahuasca ceremonies may enhance interpersonal bonds and social identity (Dobkin de Rios, 1984; Winkelman, 2007).

Another ability related to self-regulation is decentering, which engenders disidentification from internal experiences and reduced reactivity to thought content (Bernstein et al., 2019). This is an important mechanism of change that functions as an emotional regulation–mediating process underlying different salutary effects (Teasdale et al., 2002). Given the relevance of this mechanism to promote health and well-being, multiple clinical intervention programs focus on teaching patients the techniques to develop this ability (Sauer, Baer, 2010; Bieling et al., 2012). Our results show a high-effect size in the ability of decentering at all point assessments as well as a moderate correlation between the improvement in decentering and psychological well-being after the retreat. These results are supported by several studies, which have found a significant improvement in this ability, 24 h after taking ayahuasca (Soler et al., 2016; Dominguez-Clave et al., 2019; Gonzalez et al., 2020; Murphy-Beiner, Soar, 2020), being maintained over one-year follow-up (Gonzalez et al., 2020). Given that the development of this skill has not been formally taught in the ayahuasca ceremonies of those previous studies, these results lead us to hypothesize about the possibility that the development of this ability could be acquired and sustained long-term through an experiential learning process (Kolb, 1984) with what is traditionally known as “master plants” (Luna, 1986). Experiential learning is an embodied, complex process often triggered by an emotionally intense experience in a contextually rich learning environment that becomes embodied in memory traces (Morris, 2019). Understanding the benefits of ayahuasca as an experiential learning process opens the door to investigating multiple mechanisms of action that have not yet been studied in depth in the field of psychedelics, such as the mediating role of cognitive reappraisal and contextualized meaning-making (Zepke and Leach, 2002).

It is important to consider the limitations of this study. First, the naturalistic design of the study does not allow us to isolate the effects of ayahuasca from the other diverse variables in the setting, which include the work of the Shipibo *onanyabo* during the ayahuasca ceremony. In fact, the separation of the effects of ayahuasca from the *onanyabo’s* work is not conceived within this traditional medicine system. Moreover, without a placebo control group, it is not possible to determine the efficacy of ayahuasca as herbal medicine to promote well-being. Second, the sample selected is not representative of the general population as they must have financial resources to attend this center, and all of them wish to drink ayahuasca motivated by personal or spiritual growth at baseline. Third, since participation in the study was not mandatory for people who signed up for the retreats, not all those who attended the center during the two-year period were recruited for the study. Furthermore, social desirability response bias is associated with the use of self-report questionnaires.

*Vegetalismo*, which includes the use of ayahuasca and many other plant medicines, is still the primary operational health-care system in the Peruvian jungle since it is empirically efficient for the people, deeply rooted in the culture, and financially accessible (Mabit, 1993). At the same time, more and more Western people travel to the Peruvian cities, such as Iquitos, in search of other alternative forms of healing and self-transformation they do not find in the Western biomedical system (Fotiou, 2010; Jara, 2014). However, in the absence of international regulation of this medical system, people are exposed to several risks; these risks were mentioned by 92% of the sample. Enhancing well-being is the best way to promote mental health (Hupper, 2009) and the vision of the World Health Organization is the attainment of the highest possible level of health by all people (WHO, 2020a). In view of this, we hope that this study can contribute to the further recognition of ayahuasca as a herbal medicine used in the traditional medical context. Future studies are needed to explore and evaluate *vegetalismo* as a traditional medicine system.

## Data Availability

The original contributions presented in the study are included in the article/Supplementary Material, further inquiries can be directed to the corresponding author.

## References

[B1] Barbosa P. C. R. Strassman R. J. da Silveira D. X. Areco K. Hoy R. Pommy J. (2016). Psychological and Neuropsychological Assessment of Regular Hoasca Users. Compr. Psychiatry 71, 95–105. 10.1016/j.comppsych.2016.09.003 27653781

[B2] Bernstein A. Hadash Y. Fresco D. M. (2019). Metacognitive Processes Model of Decentering: Emerging Methods and Insights. Curr. Opin. Psychol. 28, 245–251. 10.1016/j.copsyc.2019.01.019 30908987 PMC7459352

[B3] Bieling P. J. Hawley L. L. Bloch R. T. Corcoran K. M. Levitan R. D. Young L. T. (2012). Treatment-specific Changes in Decentering Following Mindfulness-Based Cognitive Therapy versus Antidepressant Medication or Placebo for Prevention of Depressive Relapse. J. Consulting Clin. Psychol. 80, 365–372. 10.1037/a0027483 PMC3365628 22409641

[B4] Bouso J. C. González D. Fondevila S. Cutchet M. Fernández X. Ribeiro Barbosa P. C. (2012). Personality, Psychopathology, Life Attitudes and Neuropsychological Performance Among Ritual Users of Ayahuasca: a Longitudinal Study. PLoS One 7, e42421. 10.1371/journal.pone.0042421 22905130 PMC3414465

[B5] Bouso J. C. Palhano-Fontes F. Rodríguez-Fornells A. Ribeiro S. Sanches R. Crippa J. A. S. (2015). Long-term Use of Psychedelic Drugs Is Associated with Differences in Brain Structure and Personality in Humans. Eur. Neuropsychopharmacol. 25, 4. 10.1016/j.euroneuro.2015.01.008 25637267

[B6] Bustos S. (2008). The Healing Power of the Icaros: A Phenomenological Study of Ayahuasca Experiences. Dissertation. San Francisco, CA: California Institute of Integral Studies.

[B7] Callaway J. C. McKenna D. J. Grob C. S. Brito G. S. Raymon L. P. Poland R. E. (1999). Pharmacokinetics of Hoasca Alkaloids in Healthy Humans. J. Ethnopharmacol. 65, 3. 10.1016/s0378-8741(98)00168-8 10404423

[B8] Carmody J. Baer R. A. Lb Lykins E. Olendzki N. (2009). An Empirical Study of the Mechanisms of Mindfulness in a Mindfulness-Based Stress Reduction Program. J. Clin. Psychol. 65, 613–626. 10.1186/2050-7283-2-1810.1002/jclp.20579 19267330

[B9] Cohen J. (1992). A Power Primer. Psychol. Bull. 112, 155–159. 10.1037/0033-2909.112.1.155 19565683

[B10] Diener E. Suh E. M. Lucas R. E. Smith H. L. Smith H. L. (1999). Subjective Well-Being: Three Decades of Progress. Psychol. Bull. 125, 276–302. 10.1037/0033-2909.125.2.276

[B11] Dobkin de Rios M. (1984). Hallucinogens: Cross-Cultural Perspectives. Albuquerque: University of New Mexico Press.

[B12] Domínguez-Clavé E. Soler J. Pascual J. C. Elices M. Franquesa A. Valle M. (2019). Ayahuasca Improves Emotion Dysregulation in a Community Sample and in Individuals with Borderline-like Traits. Psychopharmacology 236, 573–580. 10.1007/s00213-018-5085-3 30406413

[B13] Fábregas J. M. González D. Fondevila S. Cutchet M. Fernández X. Barbosa P. C. R. (2010). Assessment of Addiction Severity Among Ritual Users of Ayahuasca. Drug and Alcohol Dependence 111, 257–261. 10.1016/j.drugalcdep.2010.03.024 20554400

[B14] Fontanilla D. Johannessen M. Hajipour A. R. Cozzi N. V. Jackson M. B. Ruoho A. E. (2009). The Hallucinogen N,N-Dimethyltryptamine (DMT) Is an Endogenous Sigma-1 Receptor Regulator. Science 323, 934–937. 10.1126/science.1166127 19213917 PMC2947205

[B15] Fotiou E. (2010). From Medicine Men to Day Trippers: Shamanic Tourism in Iquitos, Peru. Dissertation. Madison. WI: University of Wisconsin. 10.1037/e629402011-001

[B16] Franquesa A. Sainz-Cort A. Gandy S. Soler J. Alcázar-Córcoles M. Á. Bouso J. C. (2018). Psychological Variables Implied in the Therapeutic Effect of Ayahuasca: A Contextual Approach. Psychiatry Res. 264, 334–339. 10.1016/j.psychres.2018.04.012 29674223

[B17] Fresco D. M. Moore M. T. van Dulmen M. H. M. Segal Z. V. Ma S. H. Teasdale J. D. (2007). Initial Psychometric Properties of the Experiences Questionnaire: Validation of a Self-Report Measure of Decentering. Behav. Ther. 38, 234–246. 10.1016/j.beth.2006.08.003 17697849

[B18] Gergen K. J. (1985). “Social Constructionist Inquiry: Context and Implications,” in The the Social Construction of the Person. Editors Gergen K. J. Davis K. E. (New York, NY: Springer), 3–18. 10.1007/978-1-4612-5076-0_1

[B19] González D. Cantillo J. Pérez I. Farré M. Feilding A. Obiols J. E. (2020). Therapeutic Potential of Ayahuasca in Grief: a Prospective, Observational Study. Psychopharmacology 237, 1171–1182. 10.1007/s00213-019-05446-2 31938878 PMC7113212

[B20] Griffits R. R. Hurwitz E. S. Davis A. K. Johnson M. W. Jesse R. (2019). Survey of Subjective "God Encounter Experiences": Comparisons Among Naturally Occurring Experiences and Those Occasioned by the Classic Psychedelics Psilocybin, LSD, Ayahuasca, or DMT. PLoS One 14, e0214377. 10.1371/journal.pone.0214377 31013281 PMC6478303

[B21] Grob C. S. McKenna D. J. Callaway J. C. Brito G. S. NevesOberlaender E. S. G. Oberlaender G. (1996). Human Psychopharmacology of Hoasca, a Plant Hallucinogen Used in Ritual Context in Brazil. J. Nervous Ment. Dis. 184, 86–94. 10.1097/00005053-199602000-00004 8596116

[B23] Guimarães I. C. Tófoli L. F. Sussulini A. (2020). Determination of the Elemental Composition of Ayahuasca and Assessments Concerning Consumer Safety. Biol. Trace. Elem. Res. 199, 1179–1184. 10.1007/s12011-020-02226-4 32504397

[B24] Halpern J. H. Sherwood A. R. Passie T. Blackwell K. C. Ruttenber A. J. (2008). Evidence of Health and Safety in American Members of a Religion Who Use a Hallucinogenic Sacrament. Med. Sci. Monit. 14, SR15–SR22. 10.1163/1877-5888_rpp_com_025420 18668010

[B25] Hamill J. Hallak J. Dursun S. M. Baker G. (2019). Ayahuasca: Psychological and Physiologic Effects, Pharmacology and Potential Uses in Addiction and Mental Illness. Cn 17, 108–128. 10.2174/1570159X16666180125095902 PMC6343205 29366418

[B26] Hills P. Argyle M. (2002). The Oxford Happiness Questionnaire: a Compact Scale for the Measurement of Psychological Well-Being. Pers. Indiv. Differ. 33, 7. 10.1016/S0191-8869(01)00213-6

[B27] Huppert F. A. (2009). Psychological Well‐being: Evidence Regarding its Causes and Consequences. Appl. Psychol. Health Well‐being 1, 2. 10.1111/j.1758-0854.2009.01008.x

[B28] Jara V. E. (2014). Ritual and Healing: Explorations of the Placebo Phenomenon and Peruvian Curanderismo in Iquitos , Peru. Dissertation. Sarasota, FL: New College of Florida. 10.2307/j.ctvs09qcm

[B29] Jiménez-Garrido D. F. Gómez-Sousa M. Ona G. Dos Santos R. G. Hallak J. E. C. Alcázar-Córcoles M. Á. (2020). Effects of Ayahuasca on Mental Health and Quality of Life in Naïve Users: A Longitudinal and Cross-Sectional Study Combination. Sci. Rep. 10, 1. 10.1038/s41598-020-61169-x 32139811 PMC7057990

[B30] Kaptchuk T. J. (2002). The Placebo Effect in Alternative Medicine: Can the Performance of a Healing Ritual Have Clinical Significance?. Ann. Intern. Med. 136, 817. 10.7326/0003-4819-136-11-200206040-00011 12044130

[B31] Kavenská V. Simonová H. (2015). Ayahuasca Tourism: Participants in Shamanic Rituals and Their Personality Styles, Motivation, Benefits and Risks. J. Psychoactive Drugs 47, 351–359. 10.1080/02791072.2015.1094590 26514589

[B32] Labate B. C. Araújo W. S. (2004). O uso ritual da ayahuasca. Brazil: CampinasMercado de Letras.

[B33] Langdon E. J. (1979). “Yagé Among the Siona: Cultural Patterns in Visions,” in The Spirits, Shamans, and Stars: Perspectives from South America. Editors Browman D. L. Schwarz R. A. (New York, NY: The Gruyter Mouton)), 63–80.

[B34] Lawn W. Hallak J. E. Crippa J. A. Dos Santos R. Porffy L. Barratt M. J. (2017). Well-being, Problematic Alcohol Consumption and Acute Subjective Drug Effects in Past-Year Ayahuasca Users: a Large, International, Self-Selecting Online Survey. Sci. Rep*.* 7, 1. 10.1038/s41598-017-14700-6 29123145 PMC5680239

[B35] Lorimer D. (2019). The Galileo Commission: Towards a Post-materialist Science. An Invitation to Look through the Telescope. J. Study Spirituality 9, 67–72. 10.1080/20440243.2019.1581398

[B36] Luna L. E. (1986). Vegetalismo Shamanism Among the Mestizo Population of the Peruvian Amazon. Stockholm: Almqvist and Wiksell International.

[B37] Mabit J. (1993). Cuidado tradicional de la salud en la provincia de San Martín. Available at: https://www.takiwasi.com/docs/arti_esp/cuidado_tradicional_salud.pdf (Accessed September 28, 2020).

[B38] Markus H. Wurf E. (1987). The Dynamic Self-Concept: A Social Psychological Perspective. Annu. Rev. Psychol. 38, 299–337. 10.1146/annurev.ps.38.020187.001503

[B39] McKenna D. J. (1999). “Ayahuasca: an Ethnopharmacologic History,” in Ayahuasca: Hallucinogens, Consciousness, and the Spirit of Nature. Editor Metzner R. (New York, NY: Thunder‘s Mouth Press), 187–213.

[B40] Medvedev O. N. Landhuis C. E. (2018). Exploring Constructs of Well-Being, Happiness and Quality of Life. PeerJ 6, e4903. 10.7717/peerj.4903 29876148 PMC5985772

[B41] Mello S. M. Soubhia P. C. Silveira G. Corrêa-Neto N. F. Lanaro R. Costa J. L. (2019). Effect of Ritualistic Consumption of Ayahuasca on Hepatic Function in Chronic Users. J. Psychoactive Drugs 51, 3–11. 10.1080/02791072.2018.1557355 30582439

[B42] Michaelson J. Abdallah S. Steuer N. Thompson S. Marks N. Aked (2009). National Accounts of Well-Being: Bringing Real Wealth onto the Balance Sheet. Available at: http://www.nationalaccountsofwellbeing.org/learn/download-report.html (Accessed October 5, 2020).

[B43] Miller F. G. Kaptchuk T. J. (2008). The Power of Context: Reconceptualizing the Placebo Effect. J. R. Soc. Med. 101, 222–225. 10.1258/jrsm.2008.070466 18463276 PMC2376272

[B44] Morris T. H. (2019). Experiential Learning - a Systematic Review and Revision of Kolb’s Model. Interactive Learn. Environments 28, 1064–1077. 10.1080/10494820.2019.1570279

[B45] Murphy-Beiner A. Soar K. (2020). Ayahuasca's 'afterglow': Improved Mindfulness and Cognitive Flexibility in Ayahuasca Drinkers. Psychopharmacology 237, 1161–1169. 10.1007/s00213-019-05445-3 31927605

[B46] Nour M. M. Evans L. Nutt D. Carhart-Harris R. L. (2016). Ego-dissolution and Psychedelics: Validation of the Ego-Dissolution Inventory (EDI). Front. Hum. Neurosci. 10, 131. 10.3389/fnhum.2016.00269 27378878 PMC4906025

[B47] Ona G. Kohek M. Massaguer T. Gomariz A. Jiménez D. F. Dos Santos R. G. (2019). Ayahuasca and Public Health: Health Status, Psychosocial Well-Being, Lifestyle, and Coping Strategies in a Large Sample of Ritual Ayahuasca Users. J. Psychoactive Drugs 51, 2. 10.1080/02791072.2019.15679617 30732540

[B48] Osório F. D. L. Sanches R. F. Macedo L. R. Dos Santos R. G. Maia-de-Oliveira J. P. Wichert-Ana L. (2015). Antidepressant Effects of a Single Dose of Ayahuasca in Patients with Recurrent Depression: a Preliminary Report. Rev. Bras. Psiquiatr. 37, 13–20. 10.1590/1516-4446-2014-1496 25806551

[B49] Palhano-Fontes F. Barreto D. Onias H. Andrade K. C. Novaes M. M. Pessoa J. A. (2019). Rapid Antidepressant Effects of the Psychedelic Ayahuasca in Treatment-Resistant Depression: a Randomized Placebo-Controlled Trial. Psychol. Med. 49, 655–663. 10.1017/S0033291718001356 29903051 PMC6378413

[B50] Park J. Park J. (2017). Mediating Effect of Decentering on the Relationship between Self Discouragement and Psychological Wellbeing of University Students. Korean J. Str Res. 25, 52–56. 10.17547/kjsr.2017.25.1.52

[B51] Power M. Bullinger M. Harper A. (1999). The World Health Organization WHOQOL-100: Tests of the Universality of Quality of Life in 15 Different Cultural Groups Worldwide. Health 18, 5. 10.1037//0278-6133.18.5.49510.1037/0278-6133.18.5.495 10519466

[B52] Riba J. Valle M. Urbano G. Yritia M. Morte A. Barbanoj M. J. (2003). Human Pharmacology of Ayahuasca: Subjective and Cardiovascular Effects, Monoamine Metabolite Excretion, and Pharmacokinetics. J. Pharmacol. Exp. Ther. 306, 73–83. 10.1124/jpet.103.049882 12660312

[B53] Ryff C. (1989). Happiness Is Everything, or Is it? Explorations on the Meaning of Psychological Well-Being. J. Pers. Soc. Psychol. 57, 6. 10.1037/0022-3514.57.6.1069

[B54] Sanches R. F. de Lima Osório F. dos Santos R. G. Macedo L. R. H. Maia-de-Oliveira J. P. Wichert-Ana L. (2016). Antidepressant Effects of a Single Dose of Ayahuasca in Patients with Recurrent Depression. J. Clin. Psychopharmacol. 36, 77–81. 10.1097/JCP.0000000000000436 26650973

[B55] Santos R. G. D. (2011). Ayahuasca: Physiological and Subjective Effects, Comparison with D-Amphetamine, and Repeated Dose Assessment. Spain: Universitat Autònoma de Barcelona.

[B56] Sauer S. Baer R. A. (2010). “Mindfulness and Decentering as Mechanisms of Change in Mindfulness-And Acceptance-Based Interventions,” in The Assessing Mindfulness and Acceptance Processes in Clients: Illuminating the Theory and Practice of Change. Editor Baer R. A. (Oakland, CA: Context Press/New Harbinger Publications), 25–50.

[B57] Schultes R. E. Hofmann A. (1992). Plants of the Gods: Their Sacred, Healing, and Hallucinogenic Powers. Rochester: Healing Arts Press.

[B58] Soler J. Elices M. Franquesa A. Barker S. Friedlander P. Feilding A. (2016). Exploring the Therapeutic Potential of Ayahuasca: Acute Intake Increases Mindfulness-Related Capacities. Psychopharmacology 233, 823–829. 10.1007/s00213-015-4162-0 26612618

[B59] Teasdale J. D. Moore R. G. Hayhurst H. Pope M. Williams S. Segal Z. V. (2002). Metacognitive Awareness and Prevention of Relapse in Depression: Empirical Evidence. J. Consult. Clin. Psychol. 70, 2. 10.1037//0022-006x.70.2.275 11952186

[B60] Temple of the Way of Light. (2019). Medical Guidelines. Available at: https://templeofthewayoflight.org/integrating-ayahuasca/medicalguidelines/ (Accessed October 04, 2019).

[B61] Temple of the Way of Light. (2016). The Temple of the Way of Light. Available at: https://templeofthewayoflight.org (Accessed July 09, 2018).

[B62] Tupper K. W. (2009). Ayahuasca Healing beyond the Amazon: The Globalization of a Traditional Indigenous Entheogenic Practice. Glob. Netw. 9, 1. 10.1111/j.1471-0374.2009.00245x

[B63] Tupper K. W. (2008). The Globalization of Ayahuasca: Harm Reduction or Benefit Maximization?. Int. J. Drug Pol. 19, 297–303. 10.1016/j.drugpo.2006.11.001 18638702

[B64] Turner E. (1992). “Training to See what the Natives See,” in The Shaman’s through Time: 500 Years on the Path of Knowledge. Editors Narby J. Huxley F. (London, United Kingdom: Thames & Hudson), 260–262.

[B65] WHOQOL Group (1998). Development of the World Health Organization WHOQOL-BREF Quality of Life Assessment. The WHOQOL Group. Psychol. Med. 28, 551–558. 10.1017/s0033291798006667 9626712

[B66] WHOQOL SRPB Group (2006). A Cross-Cultural Study of Spirituality, Religion, and Personal Beliefs as Components of Quality of Life. Soc. Sci. Med. 62, 1486–1497. 10.1016/j.socscimed.2005.08.001 16168541

[B67] Winkelman M. (2007). “Shamanic Guidelines for Psychedelic Medicine,”. The Psychedelic Medicine—New Evidence for Hallucinogenic Substances as Treatments. Editors Winkelman M. Roberts T. (Praeger: Westport)), 143–167.

[B69] World Health Organization. (2006). Constitution of the World Health Organization. Available at: https://www.who.int/governance/eb/who_constitution_en.pdf (Accessed September 28, 2020).

[B70] World Health Organization. (2020c). Mental Health. Available at: https://www.who.int/news-room/ (Accessed September 28, 2020).

[B71] World Health Organization. (2020a). Our Values. Available at: https://www.who.int/about/who-we-are/our-values (Accessed October 5, 2020)

[B72] World Health Organization. (2010). Regional Office for South-East AsiaTraditional Herbal Remedies for Primary Health Care. Available at: https://apps.who.int/iris/handle/10665/206024 (Accessed September 28, 2020).

[B73] World Health Organization. (2001). The World Health Report 2001: Mental Health: New Understanding, New Hope. Available at: https://apps.who.int/iris/handle/10665/42390 (Accessed October 5, 2020).

[B74] World Health Organization. (2020b). Traditional, Complementary and Integrative Medicine. Available at: https://www.who.int/health-topics/traditional-complementary-and-integrative-medicine#tab=tab_1 (Accessed September 28, 2020)

[B75] World Health Organization. (2018). WHO Releases New International Classification of Diseases (ICD 11). Available at: https://www.who.int/news- Available at: room/detail/18-06-2018-who-releases-new-international-classification-of-diseases-(icd-11 (Accessed September 28, 2020)

[B76] World Health Organization. (2013). WHO Traditional Medicine Strategy: 2014–2023. Available at: https://apps.who.int/iris/bitstream/handle/10665/92455/9786167697581-tha.pdf (Accessed September 28, 2020).

[B77] Zepke N. Leach L. (2002). Contextualised Meaning Making: One Way of Rethinking Experiential Learning and Self-Directed Learning?. Stud. Contin. Educ. 24, 2. 10.1080/10494820.2019.157027910.1080/0158037022000020992

[B78] Zuluaga G. (2000). El pensamiento de los mayores. Código de Ética de la Medicina Indígena del Piedemonte Amazónico Colombiano. Available at: https://umiyac.org/wp-content/uploads/2019/11/el_pensamiento.pdf (Accessed September 28, 2020).

