# Supplementary material for: The Shipibo Ceremonial Use of Ayahuasca to Promote Well-Being: An Observational Study
Source: Front Pharmacol. 2021 May 5;12:623923. doi: 10.3389/fphar.2021.623923 (PMC8131827; doi:10.3389/fphar.2021.623923)
Supplement: Supplementary file 1 [file Table1.DOCX]

|  | Measures | T4 - T1 |  |
| --- | --- | --- | --- |
|  |  | (N=94) |  |
|  | | Mean (SD) | Sig. |
| **Psychological well-being (PWBS)** | |  |  |
|  | Autonomy | 0.52 (3.90) | 0.180 |
|  | Environmental mastery | 0.20 (3.93) | 0.159 |
|  | Personal Growth | 0.17 (2.75) | 0.684 |
|  | Positive Relations | -0.43 (4.12) | 0.933 |
|  | Purpose in life | -0.08 (4.28) | 0.895 |
|  | Self-acceptance | -0.16 (4.68) | 0.841 |
|  | Total | 0.62 (17.83) | 0.354 |
| **WHOQoL** | |  |  |
|  | Physical health | -0.23 (1.60) | 0.263 |
|  | Psychological | -0.33 (1.79) | 0.131 |
|  | Social relationships | -0.09 (2.70) | 0.390 |
| **Oxford happiness Questionnaire** | | 0.03 (0.64) | 0.197 |
| **EQ-Decentering** | | -0.63 (5.75) | 0.343 |
| **Spirituality (WHO-QoL-SRPB)** | |  |  |
|  | Spiritual connection | 0.11 (1.04) | 0.288 |
|  | Meaning of life | -0.01 (0.67) | 0.543 |
|  | Experience of awe | 0.04 (0.69) | 0.254 |
|  | Integrity and Integration | 0.01 (0.55) | 0.525 |
|  | Spiritual strenght | -0.20 (0.57) | 0.001 |
|  | Inner peace | -0.02 (0.59) | 0.819 |
|  | Hope and optimism | -0.07 (0.55) | 0.200 |
|  | Faith | -0.05 (0.57) | 0.731 |
|  | Total | -0.02 (0.50) | 0.845 |

**S 1:** Outcome of repeated measures at post-assessment (T1) and 12 months follow-up (T4). Values are given as means differences (with standard deviations).
